# Direct and indirect therapeutic effect of traditional Chinese medicine as an add-on for non-proliferative diabetic retinopathy: a systematic review and meta-analysis

**DOI:** 10.1186/s13020-020-00380-4

**Published:** 2020-09-17

**Authors:** Xuedong An, De Jin, LiYun Duan, Shenghui Zhao, Rongrong Zhou, Fengmei Lian, Xiaolin Tong

**Affiliations:** 1 Department of Endocrinology, Guang’anmen Hospital, China Academy of Chinese Medical Sciences, Beijing, 100053 China; 2 China Academy of Chinese Medical Sciences, Beijing, 100700 China; 3 Beijing University of Chinese Medicine, Beijing, 100029 China

**Keywords:** Traditional Chinese medicine, Additional drugs, Non-proliferative diabetic retinopathy, Meta-analysis, Direct treatment, Indirect treatment

## Abstract

**Background:**

Diabetic retinopathy (DR) is the leading cause of blindness in many countries. The current treatment for non-proliferative DR (NPDR) using Western medicine (WM) alone is insufficient. At present, the combination of NPDR treatment with traditional Chinese medicine (TCM) and WM is universally applied. We aimed to evaluate the effectiveness and safety of TCM as an add-on for NPDR using a systematic review and meta-analysis.

**Method:**

Data from randomized controlled trials (RCTs) of TCM for NPDR treatment along with WM before July 6, 2019, were collected from the *China National Knowledge Infrastructure*, *Wanfang Database*, *China Biomedical Database*, *Pubmed*, *Embase,* and *Cochrane Library*. Relevant data were extracted by two reviewers. *I*^2^ statistics was adopted to appraise heterogeneity. If *I*^2^ < 50% the fixed-effects model was employed, otherwise a random-effect model was employed. (PROSPERO: CRD42019134947)

**Result:**

Eighteen RCTs (1522 patients) were included based on the inclusion and exclusion criteria. The results showed that compared with WM alone, TCM (including Compound Xueshuantong Capsule, Qiming Granule, and others) combined with WM for NPDR could improve the overall effiicacy [n = 1686, RR 1.24 (1.18,1.30), *P* < 0.00001, *I*^2^ = 0%], and reduce the influence of risk factors related to NPDR, such as glycated hemoglobin level [n = 360, MD − 0.85 (− 1.28, − 0.41), *P* = 0.0001, *I*^2^ = 72%], triglyceride (P < 0.00001), and total cholesterol (*P* = 0.0008). Moreover, no serious adverse events were reported.

**Conclusion:**

Compared with WM alone, TCM + WM could significantly improve NPDR and also reduce the correlation levels of risk factors, such as hyperglycemia, dyslipidemia. However, the small sample included in the study might lead to a publication bias, and therefore, our results should be treated with caution.

## Background

According to the International Diabetes Federation, the number of patients with diabetes mellitus (DM) worldwide has reached 415 million, and by 2040, the total number of patients will exceed 600 million. In China, the prevalence rate of DM has increased from 0.67% in 1980 to 10.4% in 2013 [1], and complications related to DM will lead to greater economic and social burden in the future. Complications of DM include macrovascular complications (i.e. cardiovascular disease, stroke), microvascular complications (i.e. diabetic nephropathy, diabetic retinopathy (DR), and diabetic peripheral neuropathy). Among them, DR is a continuous process of microcirculation and lesion development. According to the American Academy of Ophthalmology Clinical Guidelines in 2006, DR is mainly divided into non-obvious DR, non-proliferative DR (NPDR), and proliferative DR (PDR), and is often accompanied by diabetic macular edema (DME). The quality of life, psychology, and social behavior are impaired in patients with PDR, incurring increased medical expenses [2]. Vision loss can also occur in the late stage of DME or PDR, with DR being one of the major causes of blindness in many countries [3].

There are many factors involved in the pathogenesis of DR: disease course, family inheritance, hyperglycemia, hyperlipemia, or hypertension [4, 5]. For the treatment of DR, the most important factor is lowering of blood sugar levels. Studies have shown that when glycosylated hemoglobin (HbAlc) is reduced by 10% from baseline (such as 10% to 9%), the progression of DR is reduced by 43% [6]. As demonstrated by the United Kingdom Prospective Diabetes Study, patients with tightly controlled blood pressure have a significant protective effect on DR progression [7]. Hyperlipidemia is associated with an increased risk of DR in Chinese patients with T2DM, suggesting that control of blood lipids may reduce the risk of DR [8]. In the non-proliferative phase, the main treatments are oral medications, including calcium dobesilate (CD), intestinal kininogenase, and large doses of compound danshen dripping pills, while in the proliferative phase, laser surgery, and anti-vascular endothelial growth factor (VEGF) are needed. However, clinical research shows that current treatment, such as oral WM, still have certain drawbacks, are not suitable for all patients, and the effects are not significant. Laser surgery is a destructive treatment that only prevents the occurrence of blindness but does not improve the vision and fundus lesions in general, despite a recent trial that showed improvement of vision in some patients [9]. Even after VEGF injections, a relatively high proportion of patients (46%) may still require local or grid laser treatment [10].

Traditional Chinese medicine (TCM) has been used for prevention and treatment of chronic diseases for nearly 2000 years and has an indelible contribution. Significant progress has been made in the treatment of DM and its complications. Many studies have shown that Jiangtang Tiaozhi Fang can effectively reduce the levels of blood sugar and lipids [11]. Further, Compound Danshen Dripping Pills are used to treat NPDR [12]. Nowadays, the combination of TCM and WM is more common in clinical practice, which is also applied to NPDR. Randomized clinical trials (RCTs) have shown that this combination is feasible and has good curative effects [12]. However, there is currently no systematic review to prove its safety and effectiveness, and there is still a lack of high-level evidence. Therefore, we systematically evaluated the efficacy and safety of TCM as an additional drug (add-on) for the treatment of NPDR, in order to provide high-level, referenceable evidence for the selection of clinical drugs.

## Methods

This study was conducted and reported in accordance with the Preferred Reporting Project (PRISMA) guidelines for systematic reviews and meta-analysis [13]. The PROSPERO registration number is CRD42019134947.

### Search strategy and data organization

The *Chinese Knowledge Network, Wanfang Database, China Biomedical Database, Pubmed, Embase,* and *Cochrane Library* were searched for RCTs of TCM for the treatment of NPDR in combination with WM before July 6, 2019.

The search used a combined text and MeSH heading search strategy, and the search terms included "early or non-proliferative phase" and "diabetic retinopathy" and "randomized controlled trial or randomized".

Search Strategy: ((((((Non proliferative[Title/Abstract]) OR Non-proliferative [Title/ Abstract]) OR early[Title/Abstract])) AND ((((("Diabetic Retinopathy"[Mesh]) OR Diabetic Retinopathy[Title/Abstract]) OR Diabetic Retinopathies[Title/Abstract]) OR Retinopathies, Diabetic[Title/Abstract]) OR Retinopathy, Diabetic[Title/Abstract]))) AND (randomized controlled trial[Publication Type] OR randomized[Title/Abstract] OR placebo[Title/Abstract]).

The titles and abstracts included in this work were screened by Xuedong An and Fengmei Lian, respectively. Differences were resolved through discussion.

### Inclusion criteria

Studies were included if they met the following PICO(S) (participants, intervention, comparators, outcomes (study designs)) criteria:

1. Participants: The participants had NPDR; no gender and race restrictions.
2. Intervention: The TCM + WM group was treated with TCM on the basis of WM.
3. Comparator: The WM group was treated with WM.
4. Outcome: Direct outcomes included overall efficacy, visual acuity, and retinal fundus examination. Indirect outcomes included fasting blood glucose, 2-h postprandial blood glucose, glycosylated hemoglobin, triglyceride, total cholesterol, high-density lipoprotein, and low-density lipoprotein levels. The safety outcome was adverse events.
5. Study design: The study type was a randomized controlled trial (blinded or non-blinded).

Clinical studies were independently evaluated by two investigators (Xuedong An and Fengmei Lian) for methods (e.g., correct randomization), interventions, patient selection, efficacy and safety outcomes, to ensure that the studies were eligible for inclusion in our study.

### Exclusion criteria

Studies were excluded for: duplicate publication, only abstract or lack of data available, and inability to obtain full-text articles or to extract data for research.

### Data extraction

Data, including basic information such as gender, age, duration of disease, basic treatment, major outcome indicators, medication, intervention time, case shedding, and adverse events were collected by Xuedong An and Fengmei Lian.

### Assessing the risk of bias and the quality of evidence

The RCTs included in this review were assessed as low, high, or unclear risk of bias using the Cochrane Bias Risk Tool (CRBT), which included random sequence generation, allocation concealment, blinding, incomplete data, selective reporting, and other biases. Xuedong An and Fengmei Lian independently applied the CRBT to assess the risk of bias in each study. Controversial opinions were resolved through discussion.

### Statistical analysis of data

All results were analyzed using RevMan 5.2 software provided by the Cochrane Collaboration [14]. The aggregated continuous variable results were analyzed by the mean difference (MD) and 95% confidence interval (CI); the results were summarized and analyzed by relative risk (RR) and 95% CI, while I^2^ statistics were used to assess heterogeneity. If *I*^2^ ≤ 50%, the fixed effect model was used, otherwise the random effect model was employed. In addition, if the primary outcome data were missing or the trial was incomplete, the corresponding author was contacted. We also used the funnel plot to assess potential publication bias according to the Cochrane Handbook [15].

## Results

### Basic research and quality evaluation

**Fig. 1:**
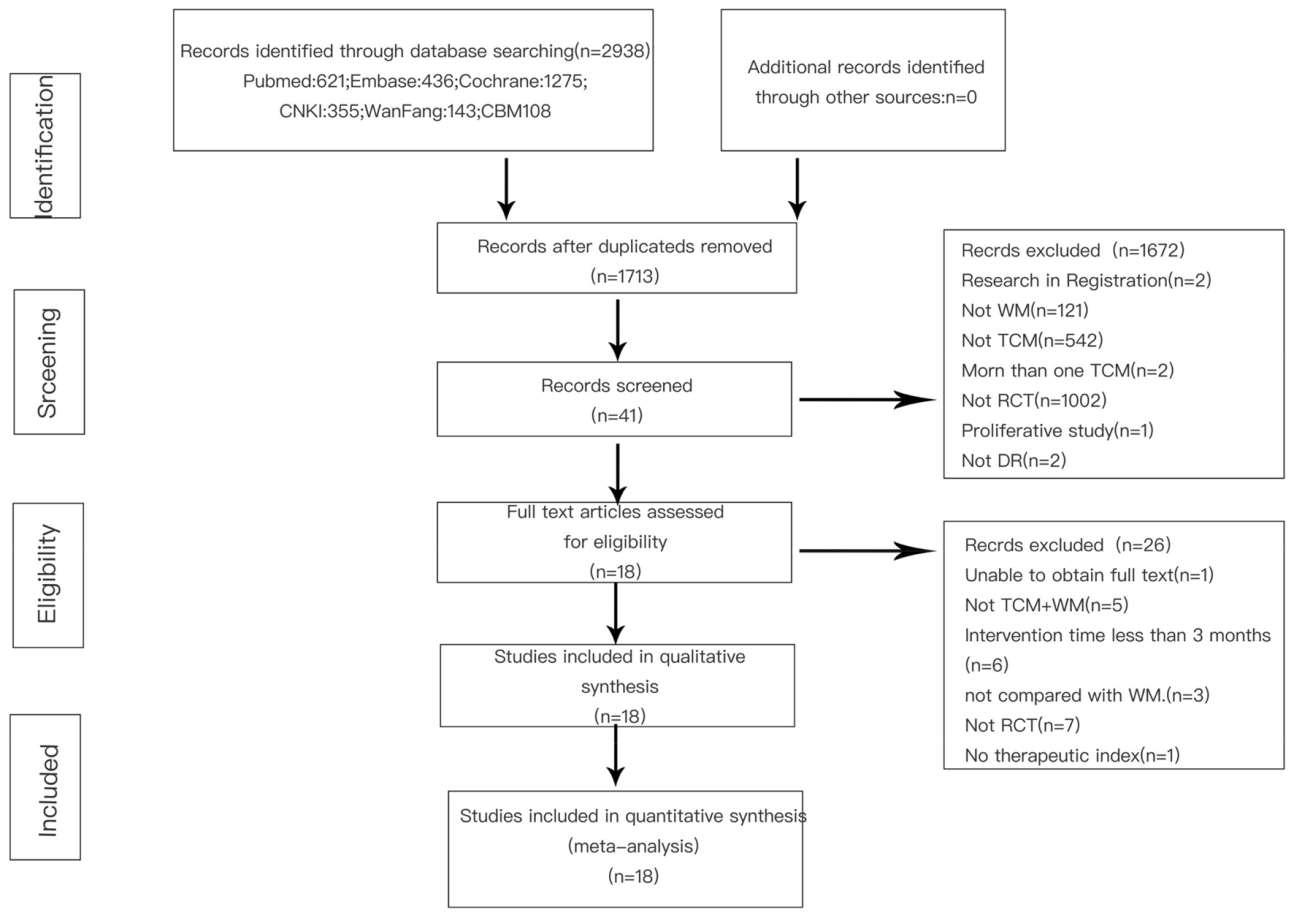
The study screening process

In the literature search, 2938 potentially related articles were found (*PubMed*: 621, *Embase*: 436, *Cochrane*: 1275, *China Knowledge Network*: 355, *Wanfang Database*: 143, *Chinese Medical Database*: 108) (Fig. 1).

**Fig. 2:**
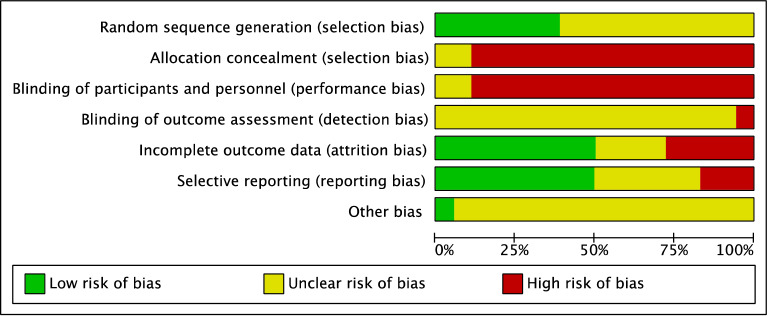
Quality assessment of the included trials-Risk of bias graph

**Fig. 3:**
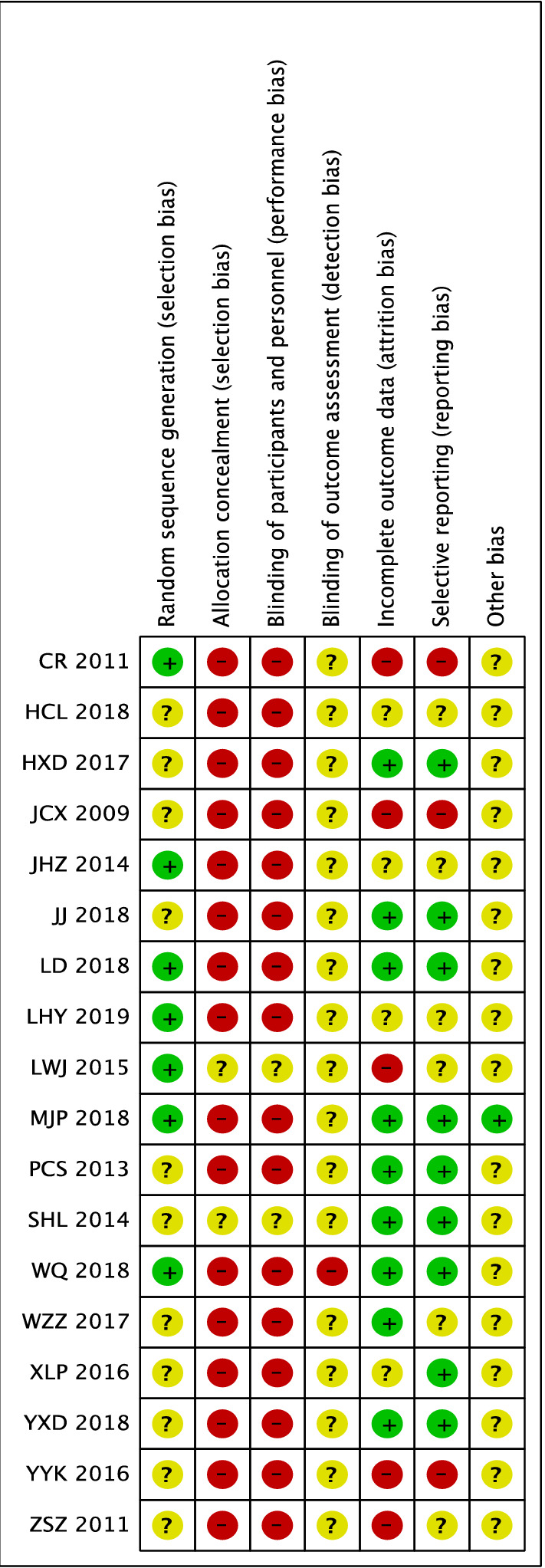
Quality assessment of included trials-Risk of bias summary

Finally, 18 studies met the inclusion criteria for this review [16–33], including 1522 patients (763 in the TCM + WM group and 759 in the WM group). Commonly used WM are CD and Yinxingdamo Injection, and commonly used TCM include Qiming Granule, and Compound Xueshuantong Capsule (Table 1). A total of 16 studies reported total efficacy [16–25, 27, 29–33], seven reported vision [17, 19, 20, 23, 26, 29, 31], four, fundus efficacy [17, 26, 28, 29], five, fasting blood glucose (FBG) [17, 26, 28, 29, 33], three reported 2 h-blood glucose (2hPG) [26, 29, 33], five, HbAlc [17, 26, 28, 29, 33], four, triglycerides (TG) [17, 26, 28, 29], five, total cholesterol (TC) [17, 26, 28, 29, 31], three, high density lipoprotein (HDL) [17, 28, 31], and five, low-density lipoprotein (LDL) [17, 26, 28, 29, 31]. Randomization was mentioned in all studies, but only seven studies showed how to generate random distribution sequences [16, 18, 19, 22, 23, 30, 33]. None of the studies presented information related to allocation hiding and blinding methods (Figs. 2, 3).

**Table 1:** Basic study characteristics

Study	Intervention duration	Main indicators	Combined treatment	Group	Number (Number of eyes)	Gender M/F	Age	DR staging (I/II/III)	Drug (dose)	Case exfoliation
JJ, 2018 [26]	12 w	Visual acuity, fundus, blood sugar, blood lipid, inflammatory index	Hypoglycemia, hypotension, lipid regulation	WM + TCM	30	14/16	58.97	–	CD (500 mg, tid) + Yangyin Xiaoyu Mingmu Prescription(100 ml, bid)	–
				WM	30	15/15	59.2	–	CD (500 mg, tid)	–
LWJ, 2015 [23]	9 m	Visual acuity, symptom score	–	WM + TCM	38	32/44	57.4 ± 2.8	–	CD (2 pills, tid) + Qihuang Mingmu Capsule (4 pills, tid)	–
				WM	38				CD (2 pills, tid)	–
SHL, 2014 [17]	3 m	Visual acuity, fundus, blood sugar, blood pressure, blood lipids	Control of blood sugar	WM + TCM	43 (86)	22/21	50.22 ± 14.82	–	CD (2 pills, tid) + Qiming Granule(4.5 g, tid)	–
				WM	43 (86)	23/20	50.53 ± 11.28	–	CD (2 pills, tid)	–
WQ, 2018 [22]	3 m	Clinical efficacy	–	WM + TCM	44	18/26	58.4 ± 7.5	–	CD (0.5 g,tid) + Qiming Granule (4.5 g, tid)	–
				WM	44	22/22	57.8 ± 6.2	–	CD (0.5 g, tid)	–
YXD, 2018 [31]	3 m	Visual acuity, mydriasis fundus, anterior segment examination, blood sugar, blood lipid	Exercise diet therapy, hypoglycemia	WM + TCM	50	24/26	54.63 ± 5.28	19/16/15	CD (0.5 g, tid) + Qiming Granule (1 bag, tid)	–
				WM	46	25/21	55.27 ± 5.42	18/13/15	CD (0.5 g, tid)	–
HCL, 2018 [29]	12 w	Fundus score, blood sugar, blood lipid	Diet and exercise, hypotension, lipid regulation	WM + TCM	40 (79)	–	58.95 ± 11.13	18/15/7	CD (0.5 g, tid) + Tangzhiping Prescription (0.5 agents, bid)	3
				WM	40 (78)	–	58.40 ± 9.21	17/17/6	CD (0.5 g, tid)	2
JHZ, 2014 [19]	6 m	Clinical symptoms, visual acuity, fundus, optical coherence tomography, hemorheology	Standard diet to control blood sugar	WM + TCM	51	31/20	57.8 ± 5.7	–	CD (0.5 g, tid) + Ziyin Yiqi Tongluo Recipe (100 ml, bid)	–
				WM	51	29/22	58.4 ± 6.3	–	CD (0.5 g,tid)	–
HXD, 2017 [20]	3 m	Clinical efficacy, depression of lesion improvement score, visual acuity, TCM symptom score	–	WM + TCM	40	23/17	52.35 ± 3.11	10/20/10	CD (0.5 g, tid) + Mimeng Flower decoction (0.5 agents, bid)	–
				WM	40	24/16	52.31 ± 3.07	8/22/10	CD (0.5 g, tid)	–
LD, 2018 [30]	3 m	Ophthalmic artery, Central retinal artery, Hypoxia-inducible factor-1α、Stromal cell-derived factor 1	Hypoglycemic drugs, diet and exercise	WM + TCM	45	27/16	48.34 ± 6.49	21/13/11	CD (250–500 mg, tid) + Compound Xueshuantong Capsule (3 pills, tid)	–
				WM	45	25/20	48.56 ± 7.64	20/15/10	CD (250–500 mg, tid)	–
XLP, 2016 [21]	6 m	Visual acuity, slit lamp, intraocular pressure, fundus fluorescein angiography	Hypoglycemia, hypotension, lipid regulation	WM + TCM	110 (216)	69/41	49.5 ± 5.9	-	CD (250–500 mg, tid) + Compound Xueshuantong Capsule (3 pills, tid)	–
				WM	110 (214)	68/42	50.2 ± 6.4	–	CD (250–500 mg, tid)	–
PCS, 2013 [25]	3 m	Fluorescein fundus angiography, hemorheology, visual field agent flash electroretinogram, overall efficacy	Basic treatment of diabetes mellitus	WM + TCM	28 (56)	12/16	51.7 ± 10.9	14/22/20	CD (500 mg, bid) + Liangxue Sanyu Decoction (0.5 agents, bid)	7
				WM	28(56)	13/15	49.3 ± 8.9	13/26/17	CD (500 mg, bid)	
LHY, 2019 [16]	3 m	Clinical efficacy, visual acuity	–	WM + TCM	60 (60)	34/26	49.4 ± 7.8	34/18/8	CD (1 pill, tid) + Compound Xueshuantong Capsule (3 pills, tid)	–
				WM	60 (60)	32/28	50.3 ± 7.4	34/16/10	CD (1 pill, tid)	–
CR, 2011 [18]	3 m	Clinical efficacy	Diabetic diet, hypoglycemic drugs	WM + TCM	30	23/7	50.13 ± 6.74	11/7/12	CD (500 mg, tid) + Yiqi Yangyin Huoxue Prescription (100 ml, tid)	–
				WM	30	21/9	51.57 ± 5.62	13/12/5	CD (500 mg, tid)	–
JCX, 2009 [27]	5 m-1y	Fundus examination, visual acuity	Hypoglycemia, hypotension and lipid regulation	WM + TCM	20	8/12	41–82	6/10/4	Yinxingdamo injection (20 ml, Intravenous drip) + Liuwei Dihuang Decoction	–
				WM	20	7/13	45–79	5/12/3	Yinxingdamo injection (20 ml, Intravenous drip)	–
ZSZ, 2011 [24]	4 m	Clinical efficacy	Hypoglycemia, lipid regulation, hypotension	WM + TCM	20 (38)	18/22	49.35	-	CD (500 mg, tid) + TangWangLing (0.5 agents, bid)	–
				WM	20 (40)				CD 500 mg, tid	–
YYK, 2016 [28]	3 m	Blood sugar, vision, fundus hemorrhage, exudation, microangioma	Hypoglycemia, lipid regulation, hypotension	WM + TCM	40	22/18	59.85 ± 11.00	15/28/11	CD (0.5 g, tid) + Panax Notoginseng Powder (2 g, tid)	-
				WM	40	19/21	63.83 ± 9.44	12/30/8	CD (0.5 g, tid)	–
WZZ, 2017 [32]	3 m	Average visual field sensitivity, related cytokines, efficacy, safety indicators	Scientific Dietary Exercise	WM + TCM	47 (47)	26/21	54.3 ± 4.9	14/18/15	CD (0.5 g, tid) + Qiming Granule (4.5 g, tid)	NA
				WM	47 (47)	29/18	54.5 ± 4.8	14/18/15	CD (0.5 g, tid)	–
MJP, 2018 [33]	5 m	Clinical efficacy, blood sugar, inflammatory factors	Symptomatic treatment	WM + TCM	27 (34)	16/11	53.02 ± 4.13	9/10/8	CD (3 pills, tid) + Compound Xueshuantong capsule (3 pills, tid)	–
				WM	27 (34)	15/12	53.08 ± 4.25	10/9/8	CD (3 pills, tid)	–

Intervention duration, m: month, w: week; gender M/F, M: male, F: female

### Main outcomes

#### Overall efficacy

**Fig. 4:**
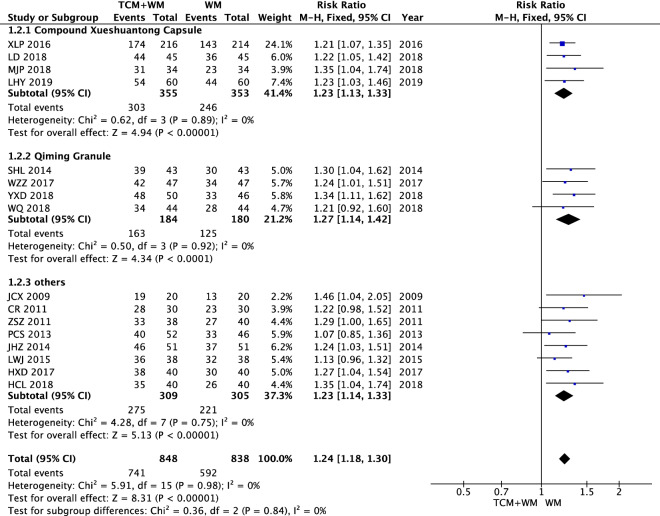
Overall efficacy

The overall efficacy in all studies showed homogeneity (*I*^2^ = 0%). Statistical data were obtained by using a fixed effect model. The results showed that the overall efficacy of TCM (including Compound Xueshuantong Capsule, Qiming Granule, and others) + WM in the treatment of NPDR was significantly better than that of WM alone [n = 1686, RR 1.24 (1.18, 1.30), *P* < 0.00001, *I*^2^ = 0%] (Fig. 4).

#### Vision

**Fig. 5:**
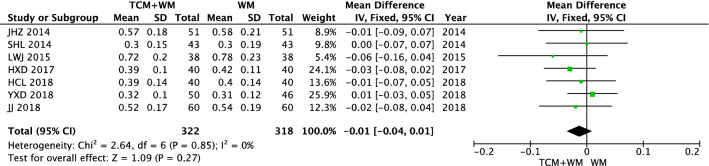
Vision before intervention

**Fig. 6:**
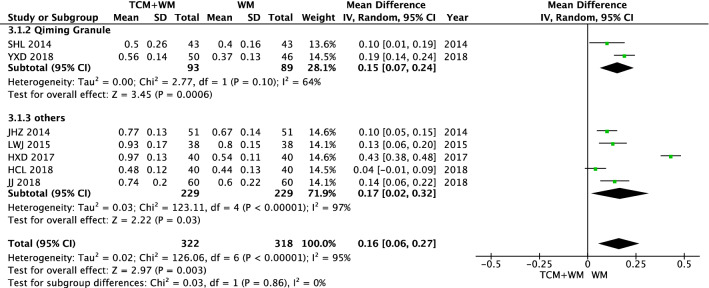
Vision after intervention

There was no difference in the vision level between the TCM + WM group and WM group before intervention (*P* < 0.27). The results of vision after intervention were heterogeneous (*I*^2^ = 95%). Data were analyzed by a random effect model. The results showed that compared with WM alone, TCM (including Qiming Granule and others) + WM treatment of NPDR significantly improved vision [n = 640, MD 0.16 (0.06, 0.27), *P* = 0.003, *I*^2^ = 95%] (Figs. 5, 6).

#### Retinal fundus

**Fig. 7:**
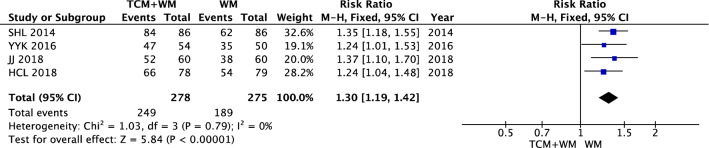
Therapeutic effect on the retinal fundus

The results showed that the retinal fundus effect showed homogeneity (*I*^2^ = 0%). Statistical data were obtained by using a fixed effect model. The results showed that compared with WM alone, TCM + WM in the treatment of NPDR fundus significantly improved [n = 553, RR 1.30 (1.19, 1.42), *P* < 0.00001, *I*^2^ = 0%] (Fig. 7).

#### FBG

**Fig. 8:**
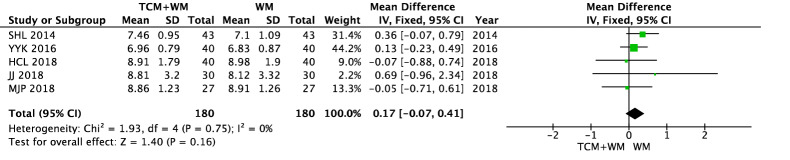
FBG before intervention

**Fig. 9:**
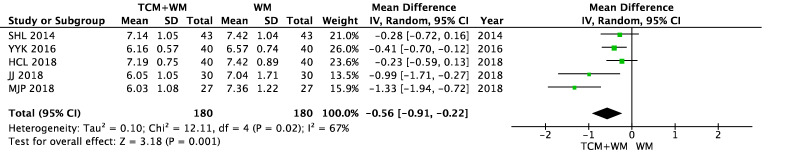
FBG after intervention

There was no difference in the FBG level between the WM group and the TCM + WM group before intervention (*P* = 0.16). The results of FBG showed heterogeneity in the two groups after intervention (*I*^2^ = 67%). Statistical data were obtained by a random effect model. The results showed that compared with WM alone, TCM + WM could effectively reduce the FBG level in patients with NPDR [n = 360, MD − 0.56 (− 0.91, − 0.22), *P* = 0.001, *I*^2^ = 67%] (Figs. 8, 9).

#### 2hPG

**Fig. 10:**
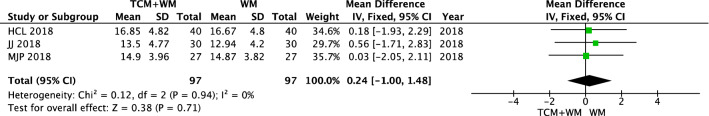
2hPG before intervention

**Fig. 11:**
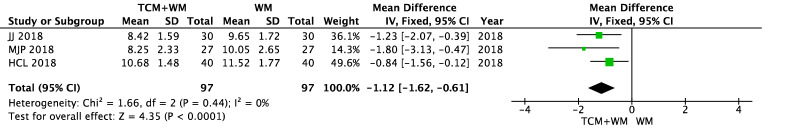
2hPG after intervention

There was no difference in the 2hPG level between the WM group and the TCM + WM group (*P* = 0.71). The results showed that the 2hPG level after intervention showed homogeneity (*I*^2^ = 0%). Statistical data were obtained by using a fixed effect model. The results showed that compared with WM alone, TCM + WM could effectively reduce the 2hPG level after intervention in patients with NPDR [n = 194, MD − 1.12 (− 1.62, − 0.61), *P* < 0.0001, *I*^2^ = 0%] (Figs. 10, 11).

#### HbAlc

**Fig. 12:**
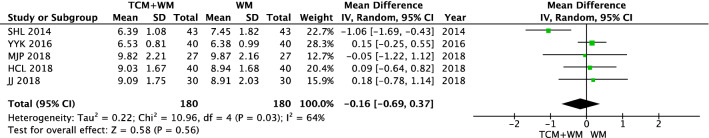
HbAlc before intervention

**Fig. 13:**
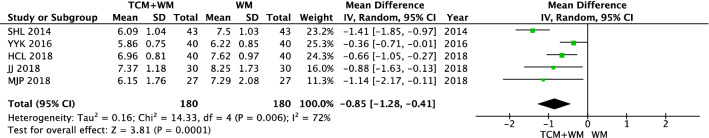
HbAlc after intervention

There was no difference in the HbAlc levels between the WM group and the TCM + WM group before intervention (*P* = 0.16). The results showed that the HbAlc level after intervention showed heterogeneity (*I*^2^ = 72%). Data were analyzed by a random effect model. The results showed that TCM + WM could effectively reduce the level of HbAlc compared with WM alone [n = 360, MD − 0.85 (− 1.28, − 0.41), *P* = 0.0001, *I*^2^ = 72%] (Figs. 12, 13).

#### TG

**Fig. 14:**
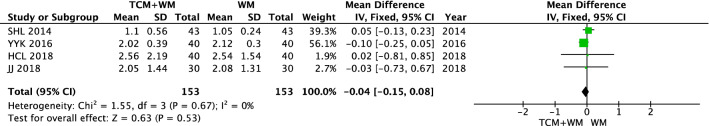
TG before intervention

**Fig. 15:**
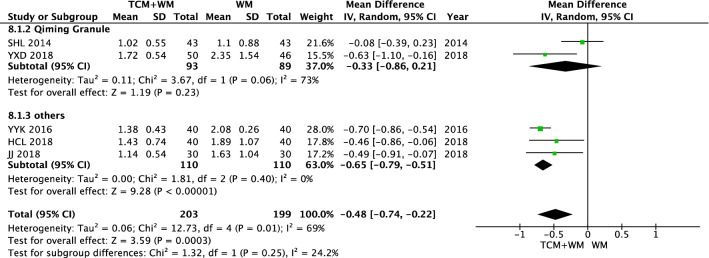
TG after Intervention

There was no difference in the TG level between the WM group and the TCM + WM group before intervention (*P* = 0.53). Studies showed that after intervention, the results of TG showed heterogeneity (*I*^2^ = 69%). A random effect model was used to analyze the data. The results showed that compared with WM alone, TCM + WM, but not Qiming Granule (*P* = 0.23), could effectively reduce TG level in patients with NPDR [n = 220, MD − 0.65 (− 0.79, − 0.51), *P* < 0.00001, *I*^2^ = 0%] (Figs. 14, 15).

#### TC

**Fig. 16:**
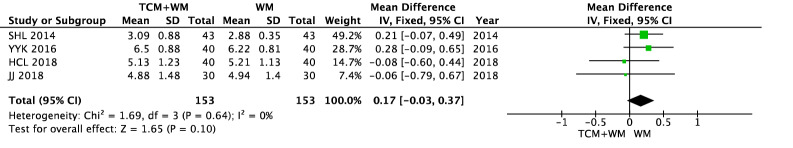
TC before intervention

**Fig. 17:**
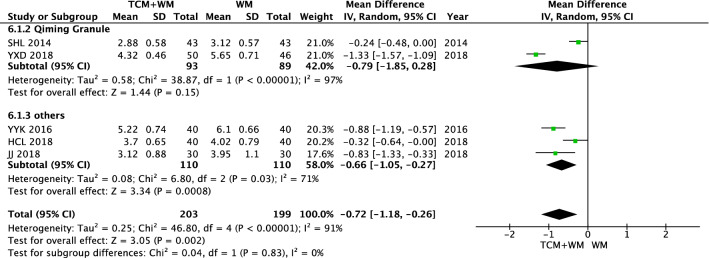
TC after intervention

There was no difference in the TC level between the WM group and the TCM + WM group before intervention (*P* = 0.10). All studies showed that TC after intervention showed heterogeneity (*I*^2^ = 91%). A random effect model was used to analyze the data. The results showed that TCM + WM could effectively reduce TC level in patients with NPDR compared with WM alone [n = 220, MD − 0.66 (− 1.05, − 0.27), *P* = 0.0008, *I*^2^ = 71%), but not Qiming Granule (*P* = 0.15) (Figs. 16, 17).

#### HDL

**Fig. 18:**
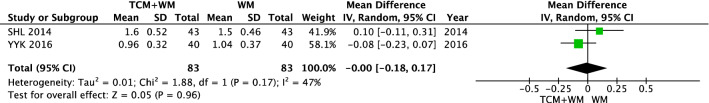
HDL before intervention

**Fig. 19:**
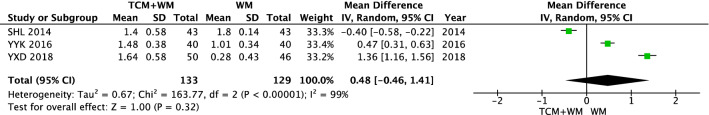
HDL after intervention

There was no difference in the HDL levels between the WM group and the TCM + WM group before intervention (*P* = 0.96). The results showed that after intervention, HDL showed heterogeneity (*I*^2^ = 99%). The data were analyzed by a random effect model. Figure 19 shows no difference in the HDL level between the WM group and the TCM + WM group after intervention [n = 262, MD 0.48 (− 0.46, 1.41), *P* = 0.32, *I*^2^ = 99%] (Figs. 18, 19).

#### LDL

**Fig. 20:**
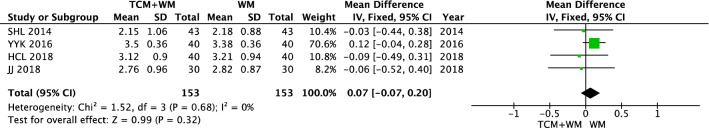
LDL before intervention

**Fig. 21:**
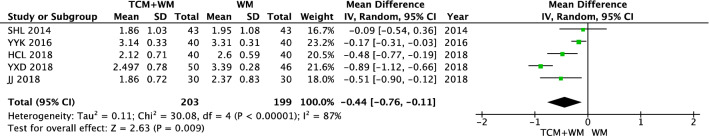
LDL after intervention

There was no difference in the LDL levels between the WM group and the TCM + WM group before intervention (*P* = 0.32). The results showed that after intervention, LDL showed heterogeneity (*I*^2^ = 87%). Statistical data were obtained by a random effect model. The results showed that compared with WM alone, TCM + WM could effectively reduce LDL levels in patients with NPDR [n = 402, MD − 0.44 (− 0.76, − 0.11), *P* = 0.009, *I*^2^ = 87%] (Figs. 20, 21).

### Adverse events

Seven studies referred to adverse events [17, 20, 21, 26, 31–33], with only one study reporting two cases of nausea and two cases of loss of appetite in the TCM + WM group, and two cases of stomach discomfort and three cases of loss of appetite in the WM group [20]. There was no difference between the two groups. No follow-up treatment for the adverse reactions was mentioned in any studies.

### Publication bias

**Fig. 22:**
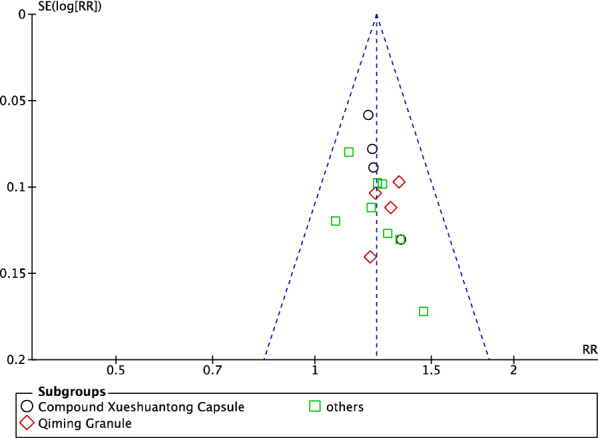
Funnel plot of total efficacy

**Fig. 23:**
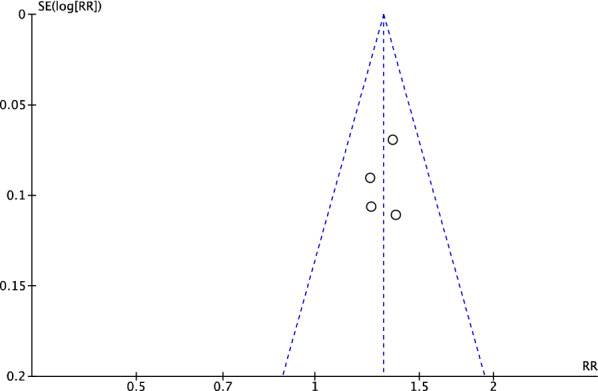
Funnel plot of fundus effect

Funnel charts were used to investigate the publication bias. The funnel charts of the overall efficacy and fundus outcomes were basically symmetrical, indicating potential publication bias. Unpublished research may be considered a factor in publication bias (Figs. 22, 23).

## Discussion

The Wisconsin Epidemiologic Study of Diabetic Retinopathy reported that approximately 75% of DM patients developed DR 10 years after diagnosis, while approximately two-thirds of patients with DR at baseline progressed to more severe DR stages, with 20% developing PDR or MDE [34]. With an increasing incidence of DM, the number of patients with DR is expected to increase from 126.6 million in 2010 to 191 million in 2030. According to current estimates, the number of DR with visual threat is expected to increase from 37.3 to 56.3 million [35]. Moreover, the cost of DR is more than half that of non-DR. To sum up, DR has resulted in a tremendous social and economic burden.

At present, the most effective intervention for DR is early screening (i.e. using fundus photography, and fundus fluorescence angiography) and early diagnosis. Studies have shown that standardized, national DR screening can reduce the blindness rate in patients with DM up to 30–50% [36]. At the same time, the DM duration, hyperglycemia and hypertension are the most relevant risk factors for DR. Previous epidemiological and clinical studies have shown that NPDR can reduce the risk and progression of DR by controlling blood sugar and blood pressure levels [37]. Strict blood pressure control can reduce the risk of DR blindness by 47% [38]. However, the current understanding of DR risk factors is still insufficient because current risk factors are not applicable to all patients [39]. For example, HbA1c may account for only 10% of DR risk, blood pressure and serum TC may account for < 10% [40], and family inheritance for about 25–50% [41]. In fact, studies have shown that DR does not occur in some patients with poor blood sugar and/or blood pressure control [42], while other patients with properly controlled blood sugar levels may have severe DR [43], suggesting that other unknown risk factors also play an important role.

In the non-proliferative phase, oral CD and pancreatic kallikrein are commonly used. CD can improve retinal microneuropathy, retinal hemorrhage, exudates, and whole blood viscosity [44], by a mechanism related to the decrease of serum endothelin-1 and high-sensitivity C-reactive protein levels [45, 46].

For PDR, treatments include laser surgery, vitrectomy, tractive retinal detachment, and injection of antiangiogenic factors or application of steroid hormones with DME [39]. Retinal photocoagulation can effectively inhibit and treat retinal neovascularization and reduce the blindness rate by 50–60% [47]. Laser surgery is a destructive treatment, which can only block blindness, but cannot improve vision or fundus lesions of patients. Anti-VEGF injection can lead to intraocular inflammation, hemorrhage, elevated intraocular pressure, and loss of retinal ganglion cells. Corticosteroid hormones prevent vascular leakage by reducing VEGF secretion and release of inflammatory cytokines. However, the incidence of corticosteroid complications is high, most commonly an increase in the intraocular pressure and cataract formation [48]. Therefore, current treatment methods cannot solve this problem with DR treatment.

Because of its simplicity, convenience, cheapness, and testing, TCM has played an indelible role in disease prevention and treatment. In the actual clinical setting, its use in combination with WM can increase the efficacy, reduce adverse events, and even reduce the dosage of WM. For the treatment of DR, Qiming Granule is commonly used, which can relieve retinal hypoxia and ischemia by increasing the retinal blood flow and improving blood circulation [49], and also lower the HbAlc level [50]; Compound Xueshuantong Capsule can protect DR by regulating the Hippo pathway [51], and reducing the VEGF expression, aldose reductase activity, whole blood viscosity and plasma viscosity [52], and lowering blood sugar levels [53].

Based on the screening criteria, 18 RCT studies were included for quality evaluation. The results show that the overall quality of research is low. Compared with WM alone, statistical results showed that TCM + WM had significant effects on clinical efficacy, visual acuity, fundus improvement, and related risk factors (i.e. blood sugar and blood lipid, but not blood pressure). Seven studies discussed the adverse events. Only one study indicated gastrointestinal discomfort, and there was no significant difference between the TCM + WM and the WM groups. No serious adverse events were reported, indicating that addition of TCM to WM for the treatment of NPDR is safe. At the same time, the results showed a great heterogeneity in the statistical analysis of visual acuity, FBG, 2hPG, HbAlC, TG, TC, HDL and LDL, probably due to factors such as fewer patients included in the study and incomplete unification of detection criteria.

Often, clinical efficacy is used to evaluate drug effects. The evaluation criteria for clinical efficacy include visual acuity, fluorescence angiography microangioma, fundus hemorrhage, exudation, edema and other symptoms. For patients with NPDR alone, clinical symptoms are mostly nor present, and they are mainly diagnosed through fundus photography, fluorescence angiography to reveal microangioma, hemorrhage spots, hard exudation, or cotton flocculent spots, while patients with PDR or DME may develop visual impairments. Vision-related findings may be present in patients with DME, but the researchers did not describe such a situation.

Funnel plots of overall efficacy and visual acuity showed basically symmetrical figures, but there was still a publication bias. In conclusion, TCM as an add-on for NPDR is effective, safe, and worthy of clinical application. However, considering the low quality of current research and possible publication bias, caution is needed.

## Conclusion

NPDR is the main microvascular complication in patients with DN and needs special attention. Based on the lack of specific treatment for NPDR, there is a need for comprehensive intervention on the disease itself and corresponding risk factors. TCM with multiple targets plays a key role in the treatment of NPDR. The results of our systematic review and meta-analysis of current high-quality clinical studies showed that compared with WM alone, TCM + WM could significantly improve NPDR and also reduce the correlation levels of risk factors such as hyperglycemia or dyslipidemia. However, considering the small sample size in the study, there is a risk of publication bias, and our results should be treated with caution. In the future, we also believe that more high-quality studies should be included to enhance the reliability of the conclusions of this study.

## Data Availability

The data included original studies and meta-analysis file with TCM + WM for NPDR. The data used to support the findings of this study are available from the corresponding author upon request.

## Abbreviations

DR: Diabetic retinopathy
NPDR: Non-proliferative diabetic retinopathy
WM: Western medicine
TCM: Traditional Chinese medicine
RCTs: Randomized controlled trials
DME: Diabetic macular edema
HbAlc: Glycosylated hemoglobin
CD: Calcium dobesilate
VEGF: Vascular endothelial growth factor
PRISMA: Preferred Reporting Project
CRBT: Cochrane Bias Risk Tool
MD: Mean difference
CI: Confidence interval
RR: Relative risk
FBG: Fasting blood glucose
2hPG: 2H-blood glucose
TG: Triglycerides
TC: Total cholesterol
HDL: High density lipoprotein
LDL: Low-density lipoprotein
